# Fracture of the Penis

**Published:** 1849-06

**Authors:** 


*Fracture of the Penis.*—A young man, native of Canton, applied to Dr. Parker for relief. He had been married about eight months. On the nuptial night, he met with insurmountable difficulty in his attempt to establish sexual intercourse with his bride, and in an effort, on that occasion, sustained a severe, and most probably, irreparable injury, which caused great pain. Since that night, erection of the penis is limited to about half an inch of its root, the extremity of the organ, with its glans, hanging flaccid.

Upon examination, a well-defined, transverse space, through the corpora cavernosa, about half an inch from the pubis, the site of fracture, was found to separate the penis into two parts.

No attempt was made to remedy this serious misfortune.—*Am. Jour. Med. Sci.*

